# An assessment of long-term sleep quality using actigraphy in survivors of critical illness

**DOI:** 10.1186/cc13600

**Published:** 2014-03-17

**Authors:** K Solverson, C Doig

**Affiliations:** 1 University of Calgary, Canada

## Introduction

Recent studies have suggested that critical illness survivors experience long-term sleep disruption [1,2]. The Actigraph device is a sleep watch that has been shown to have equivalent accuracy to polysomnography and previously used during critical illness to show sleep disruption [3]. Our objective was to assess patients long-term sleep quality using the Actigraph device.

## Methods

Study patients were selected from a 24-bed multidisciplinary ICU. Thirteen patients who were ≥18 years old, stayed longer than 4 days in the ICU and did not have and acute brain injury were followed up at 2 months post hospital discharge. The Actigraph device was given to patients to take home and worn for 72 hours. Previously validated algorithms were used to analyze sleep and wake cycles [4]. Additionally, patient completed the Pittsburgh Sleep Quality Index (PQSI), as a measure of subjective sleep quality.

## Results

Sixty-two percent of patients at 2 months post hospital discharge reported poor sleep quality as per the PSQI. The Actigraph results showed patients' average total sleep time was 6.15 hours, with a sleep efficiency of 78%. The mean time to fall asleep was 12 minutes. Patients had an average of 11 awakenings per night and were awake for an average of 7 minutes during the awakenings. There were no associations found between patients' perceived sleep quality and total sleep, sleep efficiency or sleep disruptions. Patients' severity of illness, as measured by the APACHE II score, was statistically associated with lower total sleep time (β = -12.6, *P *= 0.019), reduced sleep efficiency (β = -1.18, *P *= 0.042) and higher number of sleep disruptions (β = 0.64, *P *= 0.023). The number of days ventilated or ICU and hospital length of stay were not statistically associated with the Actigraph sleep parameters.

## Conclusion

Survivors of critical illness have high levels of sleep dysfunction as measured by actigraphy. Patients' severity of illness while critically ill appears to increase the level of long-term sleep dysfunction experienced. There is discordance between objective and subjective measures of sleep quality, which has been shown previously [5]. Objective measures of sleep quality are needed on a larger number of patients to confirm these findings.
